# Sleep to Reduce Incident Depression Effectively (STRIDE): study protocol for a randomized controlled trial comparing stepped-care cognitive-behavioral therapy for insomnia versus sleep education control to prevent major depression

**DOI:** 10.1186/s13063-022-06850-4

**Published:** 2022-12-01

**Authors:** Christopher L. Drake, David A. Kalmbach, Philip Cheng, Brian K. Ahmedani, Edward L. Peterson, Christine L. M. Joseph, Thomas Roth, Kelley M. Kidwell, Chaewon Sagong

**Affiliations:** 1 Thomas Roth Sleep Disorders & Research Center, Henry Ford Health, Detroit, MI 48202 USA; 2 Center for Health Policy & Health Services Research, Henry Ford Health, Detroit, MI 48202 USA; 3 Department of Public Health Services, Henry Ford Health, Detroit, MI 48202 USA; 4 Department of Biostatistics, University of Michigan, Ann Arbor, MI 48109 USA

**Keywords:** Insomnia, Depression, Prevention, RCT, CBT-I, Rumination

## Abstract

**Background:**

Prevention of major depressive disorder (MDD) is a public health priority. Strategies targeting individuals at elevated risk for MDD may guide effective preventive care. Insomnia is a reliable precursor to depression, preceding half of all incident and relapse cases. Thus, insomnia may serve as a useful entry point for preventing MDD. Cognitive-behavioral therapy for insomnia (CBT-I) is recommended as the first-line treatment for insomnia, but widespread implementation is limited by a shortage of trained specialists. Innovative stepped-care approaches rooted in primary care can increase access to CBT-I and reduce rates of MDD.

**Methods/design:**

We propose a large-scale stepped-care clinical trial in the primary care setting that utilizes a sequential, multiple assignment, randomized trial (SMART) design to determine the effectiveness of dCBT-I alone and in combination with clinician-led CBT-I for insomnia and the prevention of MDD incidence and relapse. Specifically, our care model uses digital CBT-I (dCBT-I) as a first-line intervention to increase care access and reduce the need for specialist resources. Our proposal also adds clinician-led CBT-I for patients who do not remit with first-line intervention and need a more personalized approach from specialty care. We will evaluate negative repetitive thinking as a potential treatment mechanism by which dCBT-I and CBT-I benefit insomnia and depression outcomes.

**Discussion:**

This project will test a highly scalable model of sleep care in a large primary care system to determine the potential for wide dissemination and implementation to address the high volume of population need for safe and effective insomnia treatment and associated prevention of depression.

**Trial registration:**

ClinicalTrials.gov NCT03322774. Registered on October 26, 2017

## Background

### Depression prevention is an urgent health priority

Major depressive disorder (MDD) surpasses asthma, diabetes, and lung cancer as a “high impact” medical condition (Medicare) and is the second most costly illness in the USA [1]. Despite decades of research refining depression treatments, many patients still do not engage in or respond to treatment. Furthermore, as each depressive episode increases the risk of MDD recurrence [2–4] and chronic debilitation, it is critical to intervene *before* an episode occurs and increases chronicity and treatment resistance. Prevention of depression, as opposed to treatment, is exponentially more impactful in reducing disease burden and needs to be the prevailing public health strategy.

Prevention efforts should target known at-risk populations based on potential mechanisms underlying risk [5]. Traditionally, studies targeting “at-risk” populations tend to treat patients with early signs of the disorder (moderate, subclinical depressive symptoms), thus limiting implications for primary prevention. Given that depression prevention is more cost-effective than treatment [5], and that experiencing MDD increases its likelihood of chronicity, there is a critical need to identify a well-defined population for prevention and to develop treatment models that are scalable and easily disseminated.

### Insomnia as a point of entry for depression prevention

While sleep disturbance is a common symptom of depression, it often occurs independently and even precedes the development of depressive symptoms. Insomnia disorder is characterized by significant difficulties falling and/or staying asleep, which occur ≥ 3 nights a week for ≥ 3 months to warrant a diagnosis [6]. Symptoms must also lead to impairments during wakefulness, including increased health-related morbidity, reduced quality of life, and impaired work performance (absenteeism, reduced productivity). Insomnia disorder is the most prevalent and burdensome sleep disorder affecting 10 to 20% of the population, with greater prevalence among women, the elderly, racial minorities, patients with comorbid psychiatric and or medical disorders, and those with low socioeconomic status [7].

A meta-analysis of > 20 studies identified insomnia as a reliable precursor of MDD such that people with insomnia are 3 times more likely to develop depression relative to good sleepers [8]. As insomnia precedes about half of incident and relapse depression cases [9, 10], insomnia can serve as an ideal entry point for depression prevention. Notably, once depressive symptoms are present, insomnia exacerbates the severity and course of depressive episodes and is the primary residual symptom during depression remission, and residual insomnia increases the likelihood of depression relapse [10–17]. Moreover, insomnia disorder is a robust risk factor for suicide [18–21]. Thus, occurrences of clinical insomnia represent critical periods throughout the trajectory of MDD that not only have important predictive value for this debilitating disorder, but also serve as opportunities for targeting a modifiable risk factor to prevent the onset and recurrence of MDD.

Cognitive behavioral therapy for insomnia (CBT-I) is the guideline-recommended treatment of choice as it has advantages regarding long-term effectiveness and safety over pharmacotherapy [22, 23]. As a standardized approach, CBT-I involves 6 modules: psychoeducation, sleep restriction, stimulus control, sleep hygiene, relaxation, and cognitive therapy. CBT-I effectiveness data have been summarized in several meta-analyses [23–26] and reviews [27–30], which conclude that CBT-I produces large improvements in sleep by substantially increasing patient-reported sleep efficiency and reducing dysfunctional beliefs about sleep.

However, the benefits of CBT-I are not limited to sleep symptoms. CBT-I has been shown to alleviate comorbid depression in insomnia patients with comorbid depression [31–35]. Additionally, and highly relevant to depression prevention, CBT-I also reduces mild or moderate (but subclinical) depressive symptoms and even depressogenic thinking with posttreatment gains durable for at least 6 months [36, 37]. These data suggest that CBT-I may protect against the development of depression via improving sleep as well as depressogenic cognitions. These findings suggest that insomnia treatment is likely to prevent MDD development and that improving sleep and reducing even mild depressive symptoms and negative cognitions may help protect against future MDD.

### Preliminary support for CBT-I in MDD prevention

Despite the possibility that treating insomnia may prevent depression, few trials have been undertaken, mostly due to the large sample sizes and long follow-up durations needed to achieve adequate power. For example, in one of the few studies to have tested depression prevention through insomnia treatment, no differences were detected, likely due to the low incidence of depression over the 6-month follow-up period [20]. Two recent randomized controlled trials (RCTs) supported depression prevention through CBT-I, but depression caseness was operationalized as clinically significant symptoms on self-report surveys [38, 39]. In another recent RCT published in 2022, CBT-I reduced the risk for clinician-diagnosed incident MDD in adults aged 60 years and older [40].

These results highlight the immense promise that insomnia therapeutics can have in MDD prevention. The next step is to examine whether insomnia treatment can reduce incidents and relapse of clinician-diagnosed MDD in a broader patient population, including young, middle, and older adults. Moreover, prevention strategies should be implemented in real-world settings where they will reach the most patients likely to benefit from this strategy.

### Barriers to MDD prevention from insomnia treatment

Despite its promise, CBT-I has limitations including an insufficient number of credentialed behavioral sleep medicine (BSM) clinicians to meet demand. Nearly 20% of US adults have insomnia [41], yet there are under 1000 board-certified BSM providers in the USA, the majority working in specialty clinics in urban areas [42]. These barriers contribute to a severe lack of access to care for millions of insomnia patients in the USA.

Finally, and quite importantly, insomnia and depression cases are typically identified and treated in primary care where access to CBT-I specialists is often limited, thereby leaving first-line behavioral options underutilized. Thus, although CBT-I effectively decreases insomnia and depression and shows promise for preventing depression, the limited availability of BSM specialists and challenges to underserved populations represent critical barriers to the scalability of clinician-led CBT-I as a first-line treatment or preventative intervention.

### Digital CBT-I as a first-line treatment

To improve access to CBT-I, web and mobile technology was utilized to develop digital CBT-I (dCBT-I). dCBT-I reduces cost and therapist time, is scalable, and empowers users to manage their own health and healthcare. dCBT-I also avoids the stigma of traditional therapy, most relevant for vulnerable populations (i.e., minority and low SES) [43]. Over a decade of numerous RCTs conclude that dCBT-I [44] is nearly as efficacious as in-person CBT-I [45].

Even so, the field struggles with its implementation, particularly in relationship with primary care, where insomnia and depression are typically identified and treated. To maximize the implementation and reach of dCBT-I, we must (1) determine the effectiveness of treating insomnia with dCBT-I when integrated into primary care and (2) evaluate its effectiveness in preventing MDD. Without such information, the therapeutic potential of dCBT-I to prevent MDD will not be realized.

Despite the success of dCBT-I, it has important limitations. Primarily, dCBT-I has limited capacity to promote treatment buy-in and to tailor specific treatment components to the needs of unique patients, potentially limiting efficacy for clinically complex cases. Thus, treatment strategies that enhance scalability while retaining the flexibility and personalization of specialty care are critically needed.

Stepped-care approaches may be ideal for delivering insomnia treatments by capitalizing on the strengths of both digital and clinician-led CBT-I treatment modalities while minimizing their disadvantages [46, 47]. Stepped-care begins with a least-restrictive intervention and then utilizes intensive specialist treatment only in patients who do not benefit initially. Our proposed stepped-care model uses dCBT-I as a least-restrictive and cost-effective first-line treatment but then follows with CBT-I with a clinician specialist only for non-remitters needing a more personalized and flexible approach after inadequately responding to digital treatment.

In a stepped-care approach for insomnia treatment, step 1 can leverage primary care and digital technology to maximize the reach of CBT-I to many patients who otherwise would have difficulty accessing care if only given the option to seek treatment in specialty clinics.

Step 2 leverages the clinical expertise and flexibility of CBT-I specialists to treat treatment-resistant patients (did not remit in step 1) who may be more clinically complex and may benefit from the more individualized approach that clinician-led CBT-I provides. A sophisticated stepped-care model integrated into primary care that capitalizes on cutting edge eHealth technology and highly trained specialty care carries immense potential for population-level depression-prevention efforts and wide dissemination.

The specific aims of the Sleep to Reduce Incident Depression Effectively (STRIDE) trial are as follows:

- Aim 1: Assess the effectiveness of stepped-care treatment for insomnia in primary care. Step 1 will involve randomization to dCBT-I or online sleep education control. dCBT-I patients who do not remit will then be re-randomized in step 2 to either clinician-led CBT-I or sleep education control.
- Aim 2: Determine the effectiveness of stepped-care insomnia treatment on the prevention of MDD. The effects of our stepped-care model on the 2-year rate of MDD incidence and relapse will be determined.
- Aim 3: Test rumination as a mediator of treatment response. Lastly, we aim to identify behaviors that facilitate treatment (mediators). Our hypothesis is that reducing rumination (operationalized here as negative repetitive thinking) will mediate the effects of dCBT-I/CBT-I on reduced insomnia symptoms and on the prevention of depression. A prior RCT showed that dCBT-I reduces ruminative thinking, which mediated nearly half of the preventive effects on developing new onset clinically significant depressive symptoms [48]. In this proposal, we will test reducing rumination as a therapeutic mechanism by which insomnia treatment reduces insomnia symptoms and the risk for MDD incidence and relapse.

## Methods/design

The present study is a large-scale stepped-care clinical trial in the primary care setting that utilizes a sequential, multiple assignment, randomized trial (SMART) design to determine the effectiveness of dCBT-I alone and in combination with clinician-led CBT-I for insomnia and the prevention of MDD incidence and relapse. The study sponsor is the National Institute of Mental Health (NIMH), which had no role in the study protocol or study design. Any proposed protocol amendments will first be approved by NIMH and then the local IRB before being implemented. Protocol deviations will be documented using a breach report form.

### Study setting

This is a RCT testing the effectiveness of a stepped-care model of insomnia treatment as implemented in primary care for the treatment of insomnia and prevention of MDD. As part of the Perfect Depression Care initiative, Henry Ford Health (HFH) routinely collects Patient Health Questionnaire (PHQ-9) [49] data from primary care patients. As part of our implementation into HFH primary care, patients who endorse sleep disturbances on the PHQ-9 will be referred to our services for further assessment and study eligibility determination. HFH primary care includes over 30 locations in southeastern Michigan.

### Patient recruitment sources

STRIDE patients are primarily recruited from multiple sources. Primarily, this study uses data from the Perfect Depression Care initiative in HFH primary care. Specifically, we identify individuals with PHQ-9 total scores below the clinical cutoff of 10 [49], but who report ≥ 1 on item #3 (“Trouble falling or staying asleep, or sleeping too much several days or more in the last 2 weeks”), which has high sensitivity (82.5%) and specificity (84.5%) for identifying insomnia symptoms in primary care. We will supplement recruitment efforts as necessary by recruiting HFH patients who have an insomnia diagnosis listed in the EMR and via study advertisements included in HFH clinics, online (e.g., HFH wellness program), in the community, and directed toward patients who previously participated in insomnia studies in our center. Interested individuals who consent online to eligibility screening will complete a battery of online surveys.

Eligibility information will be derived from electronic medical records (EMRs) and from patient reports on an online screening survey. The inclusion criteria are as follows: (1) Insomnia Severity Index (ISI) [50] score of 15 or higher to reflect clinically severe insomnia severity and (2) no clinically significant depressive symptoms as reflected by a score of 10 or lower on the Quick Inventory of Depressive Symptomatology (QIDS) [51]. The exclusion criteria are as follows: (1) age < 18 years, (2) current use of antidepressants for depression, (3) bipolar or seizure disorders, (4) untreated sleep disorders other than insomnia (e.g., obstructive sleep apnea, narcolepsy, restless legs syndrome), and (5) known diagnosis of major depression at baseline. Note that patients may start any new medication and/or therapy during participation without penalty. Eligible patients will then be randomized to step 1 treatment.

### Allocation

Patients are randomized 1:1 to CBT-I or control at each step using block randomization (step 1 uses 50-person blocks; step 2 uses six-person blocks). Only the study coordinator accesses the allocation sequence and assigns patients to groups.

### Blinding

Patients are blinded to the active therapy. Therapists are not blinded. Outcomes will be linked to a blinded group variable, which will be unblinded after primary analyses.

### Patient flow

**Fig. 1:**
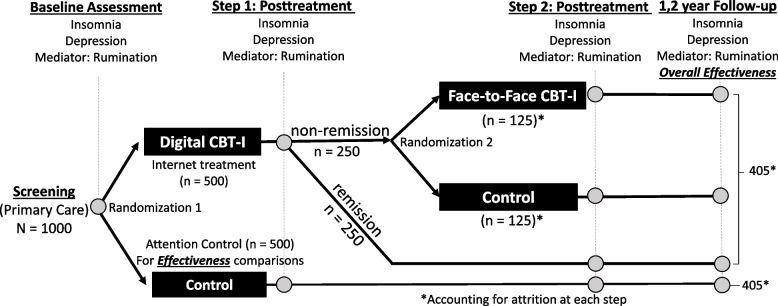
Flowchart from recruitment through treatment randomization and 2-year follow-up

See Fig. 1 for the full patient flow diagram.

#### Step 1

After baseline assessment, participants are randomized into dCBT-I or online sleep education control, each with a group size of *n* = 500. The dCBT-I group receives treatment via the Sleepio platform (www.sleepio.com), which includes all six standard components of CBT-I delivered weekly through an automated online health program. The sleep education control group receives weekly electronically delivered information regarding sleep education and sleep hygiene. Patients then complete a study outcome assessment after step 1 treatment.

#### Step 2

Patients who do not remit with dCBT-I (ISI > 7; anticipated ~ 50%) will be randomized to clinician-led CBT-I or sleep education control in step 2 of the SMART design. Treatment will be provided by personnel with CBT-I training (psychologists and nurses). Upon completing step 2, we will assess insomnia and depressive symptoms.

#### Follow-up assessments

At 1 and 2 years after the initial randomization, we will conduct follow-up assessments, which include measures of insomnia and depressive symptoms, as well as assessment of DSM-5 MDD incidence and relapse (via the SCID-5) occurring at any point in the previous 12-month period before the follow-up assessment. Rumination (aim 3) will be assessed at baseline, post-step 1, post-step 2, and at 1- and 2-year follow-ups.

### Study interventions

#### Step 1: Digital cognitive behavioral therapy for insomnia

Patients randomized to digital CBT-I in step 1 will complete the Sleepio program via the Internet (www.sleepio.com, Big Health Inc.). Sleepio is among several currently available digital CBT-I programs and was selected for this study because it is evidence-based, standardized, and fully automated. Patients are asked to complete six sessions; each session will be unlocked on a weekly basis, and patients are advised to complete one session per week. The intervention covers behavioral components (sleep restriction, stimulus control), cognitive components (e.g., cognitive restructuring, paradoxical intention), progressive muscle relaxation, and sleep hygiene. Sessions are directed by an animated “virtual therapist” who reviews and guides the progress of the patient based on the submitted sleep data. Patients complete daily sleep diaries to help monitor treatment progress and adherence to behavioral sleep strategies.

#### Step 2: Clinician-led CBT-I

As CBT-I is standardized, clinician-led CBT-I includes six standard sessions that cover the same material as digital CBT-I. However, working one-on-one with a therapist allows for greater personalization of behavioral sleep strategies and cognitive therapy, as well as personalized addressing of treatment barriers such as difficulty adhering to sleep schedules and difficulty completing homework. Patients complete daily sleep diaries to help monitor treatment progress and adherence to behavioral sleep strategies.

##### Step 1: Digital sleep education control

Patients randomized to the online sleep education condition received six weekly emails based on the National Institutes of Health guide to healthy sleep [52]. Information was provided on the basics of sleep regulation; relationships between sleep and health problems such as obesity, diabetes, and cardiovascular disease; effects of sleep-disruptive substances such as caffeine, nicotine, and alcohol; and tips on creating a sleep-conducive bedroom environment. Psychoeducation and sleep hygiene were selected because they are common in clinical practice, especially in primary care [53, 54], and also because they are commonly used as an attention control in clinical trials of insomnia. Importantly, neither sleep education nor sleep hygiene is considered an effective standalone treatment for insomnia [55].

##### Step 2: Sleep education control

Digital CBT-I non-remitters who are randomized to attention control in step 2 will receive sleep education. This includes six telemedicine sessions that cover the same material outlined above in the digital sleep education control section.

#### Data collection schedule

We will assess the study outcomes and other relevant sociodemographic and health-related information at baseline, post-step 1, post-step 2, and 1- and 2-year follow-ups. Please refer to the schedule in the study flowchart in Fig. 1. NIMH, the study sponsor, had no role in the data collection. This study does not involve collecting biological specimens.

### Study measures

Primary end-points include (aim 1) the Insomnia Severity Index (ISI) [50], (aim 2) diagnosis of major depressive disorder via clinical interview using the structured clinical interview for DSM-5 disorders [56], and (aim 3) the Pre-Sleep Arousal Scale’s cognitive factor (PSASC) to measure nocturnal rumination [57].

Screening and baseline assessment occur at the same time point and are captured using online surveys. For screening purposes, patients first complete the ISI and the Quick Inventory of Depressive Symptomatology (QIDS) [51]. Patients who endorse insomnia and deny current clinical depression will continue with the rest of the baseline assessment, which includes the Pre-Sleep Arousal Scale’s cognitive factor (PSASC) to measure nocturnal rumination in addition to sociodemographic and other health-related information.

Post-step 1 assessment occurs 1 week after completing step 1 intervention (dCBT-I or control) and includes the ISI, QIDS, and PSASC among other clinical measures. The ISI serves as our primary end-point for insomnia symptoms as tested in aim 1. Patients who report insomnia remission (operationalized as ISI ≤ 7 at post-step 1) will no longer receive insomnia treatment and will enter the follow-up data collection phase of the study described below.

On the other hand, patients who do not remit (ISI ≥ 8 at post-step 1) will then be elevated to step 2 treatment, which involves clinician-led CBT-I or sleep education control conducted via in-person or telemedicine video (the procedure was face-to-face before the COVID-19 pandemic, then switched to telemedicine video during the pandemic due to changes in clinic operations).

Post-step 2 assessment occurs 1 week after completing step 2 intervention (CBT-I or control) and includes the ISI, QIDS, and PSASC among other clinical measures. All step 2 patients then enter the follow-up data collection phase of the study.

Follow-up data collection will last for 2 years and consists of both online surveys and telemedicine video clinical interviews. Both the surveys and interviews are administered at 1 and 2 years after completing the final treatment. Surveys include the ISI, QIDS, and PSASC among other clinical symptom measures. The interviews include the SCID-5 module to diagnose major depression, which is oriented to assess over the preceding year; the MDD diagnosis serves as our primary end-point for MDD incidence and relapse for aim 2. In addition, we will assess new involvement in psychotherapy and/or pharmacotherapy for depression, which may reflect new-onset depression.

#### Data management

All data will be collected using Qualtrics and will be downloaded from their servers for analysis. Downloaded data will be anonymized and stored on secured network folders that are encrypted and password-protected to preserve confidentiality.

#### Patient retention

To maximize retention, participants will receive reminders for follow-ups via email, phone, and text using the patient portal already in use throughout our primary care networks. In addition, we will pay participants a stipend per assessment and follow up with them throughout the study to ensure participant retention, particularly during the 1- and 2-year follow-up periods. Our group has been successful in this regard (> 85% retention) in prior NIH and industry trials of > 1–2 years duration. Similar to previous long-term studies in our center, between post-treatment and each follow-up session, we will hold 2–3 raffles per year where a participant who updated their contact info during any given 4-month period wins a prize. In addition, we will send out “thank you” notes for each assessment and a regular newsletter of study progress and eventually results to those who have completed the trial.

#### Assessment of safety

CBT-I is considered safe when delivered in clinician-led and digital formats; thus, the likelihood of serious adverse events occurring during this trial is low. Even so, we will record all occurrences of serious adverse events in patients, which are defined as deaths, suicide attempts, motor vehicle collisions, and complaints about the interventions. Although CBT-I is safe, we will discontinue treatment at patient request and/or if a therapist believes CBTI to be harmful to a patient.

As insomnia increases the risk for depression and anxiety, it is possible that patients may contact the research team seeking additional mental health services. When this occurs, the research team will help guide patients to connecting with the appropriate mental health services.

Insomnia is a risk factor for suicidal thoughts. Therefore, we will regularly assess suicidal thoughts and behaviors. For patients who express imminent suicidal intent, we will guide patients to seek an evaluation in their nearest hospital emergency services and/or contact their local emergency services to perform a wellness check.

#### Data monitoring committee

An independent data monitoring committee (DMC) consisting of independent scientists was appointed. The DMC met in the first study year to review study protocols and trial safety. The DMC will then meet on an as-needed basis to review safety and trial conduct.

The steering committee is led by Drs. Drake (PI), Kalmbach (co-I), and Cheng. The steering committee, under the primary supervision of Dr. Drake, is responsible for facilitating patient recruitment, ensuring systems are in place to guarantee institutional compliance with US laws and NIH policies, reviewing trial conduct, translating the research proposal into operational plans and procedures, and overseeing the dissemination of study findings. The steering committee met weekly during the first 6 months, then biweekly thereafter to monitor the progress of the study objective, review interim analyses, and disseminate study findings. The remaining study team members, including the study coordinator and research assistants, support the study by conducting recruitment, consenting, and data collection at each site as well as data tracking in Qualtrics and reporting on progress.

#### Analytic plan

Detailed descriptive analysis of all quantitative data will be performed. We will use information from this preliminary investigation to (1) describe univariate and bivariate sample distributions of the data, (2) identify the interrelationships among variables (i.e., need for covariate adjustment), and (3) check for violation of assumptions underlying identified statistical techniques (e.g., independence, linearity, homoscedasticity, and normality). The study design involves comparing the study arms to evaluate their relative changes from pre-treatment to post-treatment. Consistent with appropriate clinical trial methodology, we will perform intent-to-treat analyses utilizing all data points with mixed modeling. We will use full information maximum likelihood estimation to handle data missingness in our mixed models. NIMH, the study sponsor, had no role in the analysis plan and will have no role in the data analysis or interpretation.

- Aim 1: The immediate focus of the analysis will be on the post-treatment impact on sleep outcomes (ISI) of the Internet-based dCBT-I, followed by the clinician-led CBT-I group, compared to the sleep hygiene control group. A linear mixed model will be used to examine the post-treatment sleep values using the baseline values as covariates. The model is flexible and will allow the inclusion of other covariates as appropriate (medication, alcohol use, caffeine, etc.).
- Aim 2: MDD prevention will be assessed using a generalized mixed-effects logistic model that compares the incidence of MDD at year 1 and 2 follow-ups across treatment conditions. Covariates will also be tested, including baseline depression (QIDS), familial history of depression, and relevant demographics (e.g., sex, race). The effectiveness of the stepped-care model will be assessed via two planned comparisons. The first will compare the rate of depression between those in the full stepped-care condition (dCBT-I to clinician-led CBT-I) and those in the step 2 control (dCBT-I to control). The second will assess the overall effectiveness of the stepped-care model by comparing MDD between those who were eligible to receive stepped-care (i.e., those randomized to dCBT-I at step 1) and those who received the sleep education control at step 1. A secondary analysis will also be conducted with QIDS scores using a linear mixed-effects model to examine the differences in depression severity.
- Aim 3 (test rumination as a mediator of CBT-I on depression): Mediation significance testing will involve three models: (1) the direct effect of treatment condition on the treatment outcome (e.g., depression), (2) the effect of the treatment condition on the proposed mediator (i.e., change in rumination from pretreatment to posttreatment; this is the α pathway), and (3) the effect of the mediator (change in rumination) on the treatment outcome (e.g., depression) while controlling for treatment condition (this is the β pathway). The indirect (i.e., mediated) effect of the predictor on the outcome variable will be tested using the product of the α and β parameter estimates. Significance testing of the indirect path will be conducted using confidence intervals estimated using the PRODCLIN method implemented in R using RMediation. 

Sample size justification and power: Using current recommendations for effectiveness trials, the current study is powered using clinically relevant effect sizes (moderate) for sleep and incident MDD [58]. We hypothesize a reduction of depression incidence of 44% consistent with our preliminary data. The sample size for the project will be determined from aim 2, the most conservative primary analysis for the proposal. For the aim 2 analysis comparing the control group to the aggregated treatment group(s) on MDD incidence or recurrence, we require 405 subjects per group to detect a decrease of 44% (9.0% vs. 16.2%) with 80% power (Table 1). The number of subjects increases to 453 per group with a similar decrease but a lower incident rate in each group (8% vs. 14.4%; Table 1). Thus, targeted enrollment for aim 2 is a minimum of 405 subjects per group (group 1: all patients who received any form of active treatment in step 1 and/or step 2 vs group 2: all patients who were randomized to control in step 1; refer to the rightmost side of Fig. 1 for reference). To achieve our target sample size, we will enroll 1000 patients in step 1. Even with 19% attrition (higher than anticipated), we will retain 405 patients in each group.

**Table 1:** Final sample sizes for a 35% decrease in depression incidence/relapse at 3 conservative levels of depression onset

CBT-I/dCBT-I	Control	*n* per group
8.0%	14.4%	*n* = 453
8.5%	15.3%	*n* = 423
9.0%	16.2%	*n* = 405

## Discussion

Insomnia often triggers MDD, precedes half of depression cases, and is an early behavioral indicator of MDD risk. Furthermore, insomnia is the most frequent residual symptom that persists following successful treatment of MDD, and insomnia is a predictor of relapse. Thus, occurrences of clinical insomnia represent critical periods throughout the trajectory of depression that not only have important predictive value for this debilitating disorder but may also serve as opportunities for intervention to prevent the onset and recurrence of depression.

As insomnia is a modifiable risk factor for depression, treating insomnia to remission may prevent depression incidence and relapse. We are testing a stepped-care model of insomnia treatment wherein patients are first treated with digital CBT-I and then step up to clinician-led specialist care for insomnia as necessary. We believe that this highly accessible, efficient, and effective insomnia treatment program has immense potential to curb depression rates in this highly vulnerable patient population.

The study results will be reported in accordance with the Standard Protocol Item: Recommendations for Interventional Trials (SPIRIT) guidelines and Consolidated Standards of Reporting Trials (CONSORT) 2010 statements. The results of this trial are expected to have an important positive impact because the demonstration of the effectiveness of a stepped-care approach to insomnia and MDD prevention will justify its wide dissemination as a scalable intervention to improve sleep and mental health outcomes in primary care settings. Importantly, this is where most insomnia treatment is initiated and where CBT-I can have the greatest impact on reducing exposure to high-risk sedative hypnotics.

## STRIDE trial status

NCT03322774 version February 7, 2022. Recruitment began on March 7, 2018. Status: recruiting. Anticipated recruitment completion date: by the end of 2023.

STRIDE World Health Organization Data Set.

Registry: ClinicalTrials.gov NCT03322774.

Date of registration: October 26, 2017.

Sources of monetary support: National Institute of Mental Health (NIMH).

Primary sponsor: NIMH

Contact for public or scientific queries: Christopher L. Drake PhD—cdrake1@hfhs.org

Public and scientific title: Sleep To Reduce Incident Depression Effectively (STRIDE)

Countries of recruitment: USA

Health condition(s) or problem(s) studies: insomnia, depression

Key inclusion and exclusion criteria: ages eligible for the study: ≥ 18 years; sexes eligible for the study: both. Inclusion: clinically significant insomnia symptoms and no clinically significant depression symptoms. Exclusion: current antidepressant use, bipolar disorder, seizure disorders, untreated sleep disorders other than insomnia, and major depression diagnosis at baseline.

Study type:

Interventional

Allocation: randomized

Intervention model: parallel assignment

Masking: single-blind (patient)

Primary purpose: prevention

Date of first enrollment: March 2018

Target sample size: 1000

Recruitment status: recruiting

Primary outcomes: prevention of major depressive disorder, insomnia remission, and mediation of depression prevention by reducing nocturnal rumination.

## Data Availability

Data and materials will be available from the PI upon reasonable request after study completion.

## Abbreviations

BSM: Behavioral sleep medicine
CBT-I: Cognitive-behavioral therapy for insomnia
dCBT-I: Digital cognitive behavioral therapy for insomnia
DSM-5: Diagnostic and Statistical Manual of Mental Disorder, Fifth Edition
EMR: Electronic medical record
HFH: Henry Ford Health
ISI: Insomnia Severity Index
MDD: Major depressive disorder
NIH: National Institutes of Health
PHQ-9: Patient Health Questionnaire-9
PSASC: Pre-Sleep Arousal Scale, Cognitive Factor
QIDS: Quick Inventory of Depressive Symptomatology
RCT: Randomized controlled trial
SCID-5: Structured Clinical Interview for DSM-5 Disorders
SMART: Sequential, Multiple Assignment, Randomized Trial
STRIDE: Sleep to Reduce Incident Depression Effectively

## References

[1] Green MJ, Benzeval M (2013). The development of socioeconomic inequalities in anxiety and depression symptoms over the lifecourse. Soc Psychiatry Psychiatr Epidemiol.

[2] Bockting CLH, Spinhoven P, Koeter MWJ, Wouters LF, Schene AH (2006). Prediction of recurrence in recurrent depression and the influence of consecutive episodes on vulnerability for depression. J Clin Psychiatry.

[3] Kessing LV, Hansen MG, Andersen PK, Angst J (2004). The predictive effect of episodes on the risk of recurrence in depressive and bipolar disorders - a life-long perspective. Acta Psychiatr Scand.

[4] Solomon DA (2000). Multiple recurrences of major depressive disorder. Am J Psychiatry.

[5] Cuijpers P, Beekman AT, Reynolds CF (2012). Preventing depression: a global priority. JAMA.

[6] American Psychiatric Association (2013). Sleep-wake disorders. Diagnostic and Statistical Manual of Mental Disorders.

[7] Hajak G, Petukhova M, Lakoma MD (2011). Days-out-of-role associated with insomnia and comorbid conditions in the America Insomnia Survey. Biol Psychiatry.

[8] Baglioni C, Battagliese G, Feige B (2011). Insomnia as a predictor of depression: a meta-analytic evaluation of longitudinal epidemiological studies. J Affect Disord.

[9] Cole MG, Dendukuri N (2003). Risk factors for depression among elderly community subjects: a systematic review and meta-analysis. Am J Psychiatry.

[10] Ohayon MM, Roth T (2003). Place of chronic insomnia in the course of depressive and anxiety disorders. J Psychiatr Res.

[11] Buysse DJ, Tu XM, Cherry CR (1999). Pretreatment REM sleep and subjective sleep quality distinguish depressed psychotherapy remitters and nonremitters. Biol Psychiatry.

[12] Dew MA, Reynolds CF, Houck PR (1997). Temporal profiles of the course of depression during treatment. Predictors of pathways toward recovery in the elderly. Arch Gen Psychiatry.

[13] Franzen PL, Buysse DJ (2008). Sleep disturbances and depression: risk relationships for subsequent depression and therapeutic implications. Dialogues Clin Neurosci.

[14] McClintock SM, Husain MM, Wisniewski SR (2011). Residual symptoms in depressed outpatients who respond by 50% but do not remit to antidepressant medication. J Clin Psychopharmacol.

[15] Ohayon MM (2007). Insomnia: a ticking clock for depression?. J Psychiatr Res.

[16] Perlis ML, Giles DE, Buysse DJ, Tu X, Kupfer DJ (1997). Self-reported sleep disturbance as a prodromal symptom in recurrent depression. J Affect Disord.

[17] Pigeon WR, Hegel M, Unutzer J (2008). Is insomnia a perpetuating factor for late-life depression in the IMPACT cohort?. Sleep..

[18] Pigeon WR, Pinquart M, Conner K (2012). Meta-analysis of sleep disturbance and suicidal thoughts and behaviors. J Clin Psychiatry.

[19] Li SX, Lam SP, Yu MWM, Zhang J, Wing YK (2010). Nocturnal sleep disturbances as a predictor of suicide attempts among psychiatric outpatients. J Clin Psychiatry.

[20] McCall WV, Blocker JN, D’Agostino R (2010). Insomnia severity is an indicator of suicidal ideation during a depression clinical trial. Sleep Med.

[21] Brower KJ, McCammon RJ, Wojnar M, Ilgen MA, Wojnar J, Valenstein M (2011). Prescription sleeping pills, insomnia, and suicidality in the National Comorbidity Survey Replication. J Clin Psychiatry.

[22] Qaseem A, Kansagara D, Forciea MA, Cooke M, Denberg TD (2016). Management of chronic insomnia disorder in adults: a clinical practice guideline from the American College of Physicians. Ann Intern Med.

[23] Edinger JD, Arnedt JT, Bertisch SM (2021). Behavioral and psychological treatments for chronic insomnia disorder in adults: an American Academy of Sleep Medicine systematic review, meta-analysis, and GRADE assessment. J Clin Sleep Med.

[24] Morin CM, Culbert JP (1994). Nonpharmacological interventions for insomnia: a meta-analysis of treatment efficacy. Am J Psychiatry.

[25] Murtagh DR, Greenwood K (1995). Identifying effective psychological treatments for insomnia: a meta-analysis. J Consult Clin Psychol.

[26] Smith MT, Perlis ML (2002). Comparative meta-analysis of pharmacotherapy and behavior therapy for persistent insomnia. Am J Psychiatry.

[27] Jansson-Frojmark M, Norell-Clarke A (2018). The cognitive treatment components and therapies of cognitive behavioral therapy for insomnia: a systematic review. Sleep Med Rev.

[28] Koffel E, Bramoweth AD, Ulmer CS (2018). Increasing access to and utilization of cognitive behavioral therapy for insomnia (CBT-I): a narrative review. J Gen Intern Med.

[29] Trauer JM, Qian MY, Doyle JS, Rajaratnam SM, Cunnington D (2015). Cognitive behavioral therapy for chronic insomnia: a systematic review and meta-analysis. Ann Intern Med.

[30] Matthews EE, Arnedt JT, McCarthy MS, Cuddihy LJ, Aloia MS (2013). Adherence to cognitive behavioral therapy for insomnia: a systematic review. Sleep Med Rev.

[31] Taylor DJ, Lichstein KL, Weinstock J, Sanford S, Temple JR (2007). A pilot study of cognitive-behavioral therapy of insomnia in people with mild depression. Behav Ther.

[32] Taylor DJ, Pruiksma KE (2014). Cognitive and behavioural therapy for insomnia (CBT-I) in psychiatric populations: a systematic review. Int Rev Psychiatry.

[33] Manber R, Edinger JD (2008). Cognitive behavioral therapy for insomnia enhances depression outcome in patients with comorbid major depressive disorder and insomnia. Sleep.

[34] Wagley JN, Rybarczyk B, Nay WT, Danish S, Lund HG (2013). Effectiveness of abbreviated CBT for insomnia in psychiatric outpatients: sleep and depression outcomes. J Clin Psychol.

[35] Cunningham JEA, Shapiro CM (2018). Cognitive behavioural therapy for insomnia (CBT-I) to treat depression: a systematic review. J Psychosom Res.

[36] Kalmbach DA, Cheng P, Arnedt JT (2019). Treating insomnia improves depression, maladaptive thinking, and hyperarousal in postmenopausal women: comparing cognitive-behavioral therapy for insomnia (CBTI), sleep restriction therapy, and sleep hygiene education. Sleep Med.

[37] Ballesio A, Devoto A, Lombardo CJS, Rhythms B (2018). Cognitive behavioural therapy for insomnia reduces ruminative thinking. Sleep Biol Rhythms.

[38] Felder JN, Epel ES, Neuhaus J, Krystal AD, Prather AA (2022). Randomized controlled trial of digital cognitive behavior therapy for prenatal insomnia symptoms: effects on postpartum insomnia and mental health. Sleep..

[39] Cheng P, Kalmbach DA, Tallent G, Joseph CL, Espie CA, Drake CL (2019). Depression prevention via digital cognitive behavioral therapy for insomnia: a randomized controlled trial. Sleep..

[40] Irwin MR, Carrillo C, Sadeghi N, Bjurstrom MF, Breen EC, Olmstead R (2022). Prevention of incident and recurrent major depression in older adults with insomnia: a randomized clinical trial. JAMA Psychiatry.

[41] Roth T, Coulouvrat C, Hajak G (2011). Prevalence and perceived health associated with insomnia based on DSM-IV-TR; international statistical classification of diseases and related health problems, tenth revision; and research diagnostic criteria/international classification of sleep disorders, criteria: results from the America insomnia survey. Biol Psychiatry.

[42] Fields BG, Schutte-Rodin S, Perlis ML, Myers M. Master’s-level practitioners as cognitive behavioral therapy for insomnia providers: an underutilized resource. J Clin Sleep Med. 2013. 10.5664/jcsm.3096. 10.5664/jcsm.3096 PMC3778184 24127157

[43] Alang SM (2015). Sociodemographic disparities associated with perceived causes of unmet need for mental health care. Psychiatr Rehabil J.

[44] Lancee J, van Straten A, Morina N, Kaldo V, Kamphuis JH (2016). Guided online or face-to-face cognitive behavioral treatment for insomnia: a randomized wait-list controlled trial. Sleep..

[45] Seyffert M, Lagisetty P, Landgraf J (2016). Internet-delivered cognitive behavioral therapy to treat insomnia: a systematic review and meta-analysis. PLoS One.

[46] Manber R, Simpson NS, Bootzin RR (2015). A step towards stepped care: delivery of CBT-I with reduced clinician time.

[47] Espie CA (2009). “Stepped care”: a health technology solution for delivering cognitive behavioral therapy as a first line insomnia treatment. Sleep..

[48] Cheng P, Kalmbach DA, Cuamatzi-Castelan A, Muragan N, Drake CL (2020). Depression prevention in digital cognitive behavioral therapy for insomnia: is rumination a mediator?. J Affect Disord.

[49] Kroenke K, Spitzer RL, Williams JB (2001). The PHQ-9: validity of a brief depression severity measure. J Gen Intern Med.

[50] Bastien CH, Vallières A, Morin CM (2001). Validation of the Insomnia Severity Index as an outcome measure for insomnia research. Sleep Med.

[51] Rush AJ, Bernstein IH, Trivedi MH (2006). An evaluation of the Quick Inventory of Depressive Symptomatology and the Hamilton Rating Scale for Depression: a sequenced treatment alternatives to relieve depression trial report. Biol Psychiatry.

[52] US National Institutes of Health. Your Guide to Healthy Sleep. Bethesda: National Institutes of Health; 2011.

[53] Irish LA, Kline CE, Gunn HE, Buysse DJ, Hall MH (2015). The role of sleep hygiene in promoting public health: a review of empirical evidence. Sleep Med Rev.

[54] Grandner MA, Chakravorty S (2017). Insomnia in primary care: misreported, mishandled, and just plain missed. J Clin Sleep Med.

[55] Morgenthaler T, Kramer M, Alessi C (2006). Practice parameters for the psychological and behavioral treatment of insomnia: an update. An American Academy of Sleep Medicine report. Sleep..

[56] First MB, Williams JBW, Karg RS, Spitzer RL. User's guide for the SCID-5-CV Structured Clinical Interview for DSM-5® disorders. Clinical version. Washington DC: American Psychiatric Publishing, Inc.; 2016

[57] Nicassio PM, Mendlowitz DR, Fussell JJ, Petras L (1985). The phenomenology of the pre-sleep state: the development of the pre-sleep arousal scale. Behav Res Ther.

[58] March J, Kraemer HC, Trivedi M (2010). What have we learned about trial design from NIMH-funded pragmatic trials?. Neuropsychopharmacology..

